# The Potential Role of Spa Therapy in Managing Frailty in Rheumatic Patients: A Scoping Review

**DOI:** 10.3390/healthcare11131899

**Published:** 2023-06-30

**Authors:** Maria Chiara Maccarone, Anna Scanu, Daniele Coraci, Stefano Masiero

**Affiliations:** 1 Department of Neuroscience, Physical Medicine and Rehabilitation School, University of Padua, Via Giustiniani 2, 35128 Padua, Italy; stef.masiero@unipd.it; 2 Neurorehabilitation Unit, Department of Neuroscience, University of Padua, 35128 Padua, Italy; anna.scanu@unipd.it (A.S.); daniele.coraci@unipd.it (D.C.); 3 Department of Women’s and Children’s Health, University of Padua, 35128 Padua, Italy

**Keywords:** spa therapy, rehabilitation, balneotherapy, osteoarthritis, fibromyalgia, rheumatoid arthritis, sarcopenia

## Abstract

Frailty is not limited to the elderly, as patients with rheumatic diseases can also experience this condition. The present scoping review aimed to investigate the possibility of using the health resort setting as an alternative location for managing rheumatic patients with frailty. The research resulted in finding several in vitro, in vivo, and clinical studies, resulting in evidence supporting the effectiveness of spa treatments in reducing pain, improving function, and managing comorbidity in rheumatic diseases. Additionally, spa treatments were demonstrated to modulate the MAPK/ERK pathway and the NF-kB pathway’s activation and to reduce proinflammatory molecules’ secretion in rheumatic diseases, thus suggesting their potential effective role in the regulation of inflammaging in frailty. Moreover, the health resort setting may offer potential resources to reduce risk factors, such as drug consumption, inactivity, and disease severity, and may serve as a setting for developing prevention protocols for frailty. Future research should explore innovative approaches, such as exercise training and early diagnostics, for the overall management of frailty in rheumatic patients in the spa setting.

## 1. Introduction

The progressive increase in life expectancy worldwide, with the global population of subjects over the age of 60 expected to double in the next 30 years [1], has determined an increase in the number of individuals requiring rehabilitative intervention. In fact, individuals of the same chronological age may vary widely in their biological age due to genetic, biochemical, and environmental factors, as well as physical, psychological, and social determinants [1,2]. An important impact on individual aging is determined by frailty, a condition defined as “a biologic syndrome of decreased reserve and resistance to stresses, caused by cumulative declines across multiple physiologic systems and resulting in vulnerability to adverse outcomes” [3]. As individuals age, a physiological reduction in organ function is inevitable. However, in frail subjects, this process is abnormal and involves numerous organs and systems, leading to a disrupted homeostatic balance and a higher risk of disease and functional loss. Frail adults experience an increased mortality rate and unfavorable health outcomes, such as falls, fractures, disability, cognitive decline, and a poor quality of life [4]. Nevertheless, according to preliminary research, frailty is not exclusively an age-related syndrome, as patients suffering from rheumatic diseases may also be more prone to developing frailty [5]. Several factors can be responsible for the increased susceptibility to frailty in rheumatic patients, including genetic and epigenetic pathways, metabolic and environmental factors, and immunological, musculoskeletal, and endocrine effects [6]. In particular, systemic and chronic inflammation has been linked to frailty in rheumatic subjects [7]. The treatment of patients with rheumatic diseases and frailty must be multidisciplinary and consider comorbidity, rather than limiting treatment to the rheumatic condition. Due to the potential high number of comorbidities and polypharmacy, non-pharmacological treatments, such as spa therapy, are becoming increasingly important for individuals with rheumatic conditions. Spa therapy is a therapeutic approach that utilizes natural resources such as thermal mineral-rich waters, mud, and gases, which are administered to patients through various methods such as bathing, drinking, and inhalation treatments [8,9]. Furthermore, spa therapy is not limited to the use of natural resources alone, as it can be complemented with several other therapeutic modalities such as exercise, massage, relaxation techniques, and more [10,11]. Among the most-popular treatments within the field of spa therapy, balneotherapy, the immersion in thermal mineral-rich waters, and peloid therapy are usually employed in musculoskeletal conditions [8,12]. Pre-clinical studies and clinical trials have shown that spa interventions can be beneficial in subjects with rheumatic diseases [13,14,15,16]. The effects of immersion and exercise in thermal mineral-rich water on symptoms and inflammation appear to be superior and longer-lasting than the effects of hydrotherapy [14,17,18,19,20]. Furthermore, preliminary studies are evaluating the effects of thermal mineral-rich water on the skin’s microbiome, since bathing in thermal water could act on certain bacteria of the skin [21]. On the other hand, mud therapy improves blood flow, connective tissue flexibility, and plasma levels of β-endorphins. Additionally, mud therapy influences the neuroimmunoendocrine system and has anti-inflammatory properties [14,16,18,22]. 

The aim of this scoping review was to investigate the effectiveness of spa therapies in managing frailty in patients with rheumatic diseases. Firstly, this paper highlights the relationships between frailty and rheumatic diseases, with a particular focus on the susceptibility of rheumatic patients to develop frailty. Secondly, we emphasize the immunomodulating mechanisms of spa therapy, which could play a significant role in managing frailty in rheumatic patients. Finally, this scoping review summarizes the clinical evidence on the effectiveness of spa therapies, including mud therapy, thermal mineral-rich baths, and exercise in mineral water, which can be utilized as therapeutic means for frailty in rheumatic patients. 

## 2. Materials and Methods

**Figure 1:**
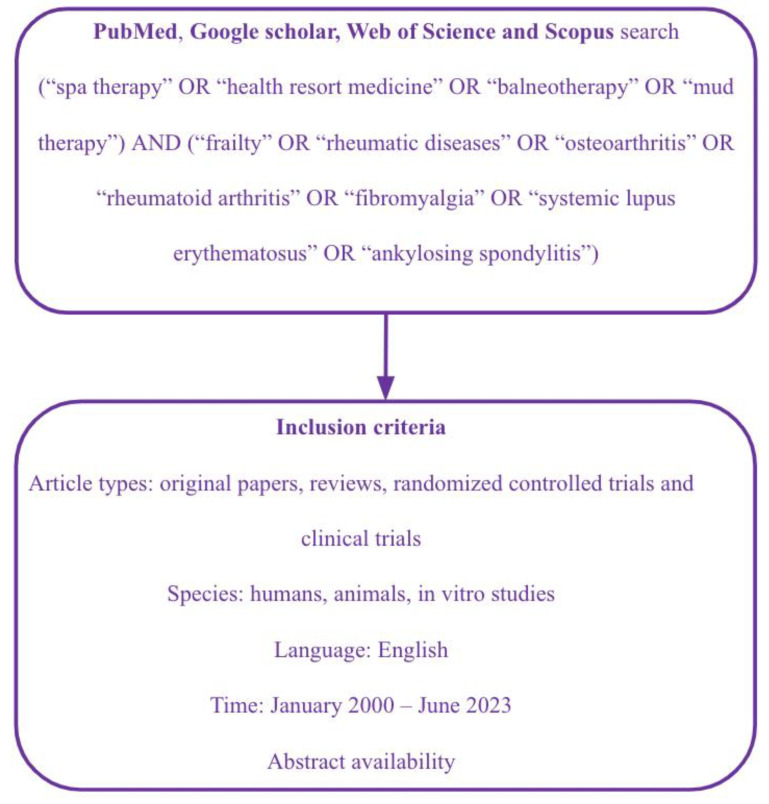
Scoping review process. RCTs: randomized controlled trials.

This scoping review followed a five-stage process based on the methodological frameworks proposed by Arksey and O’Malley and Levac et al. [23,24]. To begin, the research question was formulated by the first author in collaboration with the other authors as follows: What evidence exists regarding the impact of spa therapy on rheumatic disorders and frailty in the literature? Subsequently, a literature search was conducted on Medline (PubMed), Google Scholar, Web of Science, and Scopus by two independent researchers (M.C.M. and A.S.), using keywords such as spa therapy, health resort medicine (which includes “all medical activities originated and derived in health resorts based on scientific evidence aiming at health promotion, prevention, therapy and rehabilitation”), balneotherapy, and peloid therapy, combined with Boolean operators (Figure 1). The eligibility criteria included in vitro and in vivo research, randomized controlled trials (RCTs), or clinical trials that investigated the spa therapy interventions (balneotherapy, peloid applications, etc.) compared to another intervention or no intervention. A thorough process of identifying and selecting relevant studies was carried out. To be included in the review, studies were required to have an available abstract and be published in English. Articles with abstracts written in languages other than English were excluded. The analysis included all original research articles published from January 2000 up to June 2023 (date of last search: 27 June 2023), while case reports, letters to the editor, and studies published before 2000 were excluded. Repetitive studies and those unrelated to the topic were also excluded.

Data extraction was performed, excluding papers that did not meet the inclusion criteria. The first author and her assistants independently screened the papers and obtained full-text versions.

## 3. Frailty in Rheumatic Diseases

Frailty is a multifaceted syndrome characterized by a decrease in an individual’s reserve and resistance to stressors. This decrease is a result of cumulative declines across multiple physiological systems, and it can make an individual vulnerable to adverse outcomes [3]. Several criteria have been suggested for defining frailty, which include low physical activity, muscle weakness, slowed walking speed, self-reported weariness, and accidental weight loss [3,25]. Pre-frailty status is defined by the presence of one to two of the aforementioned criteria, while frailty is defined as the presence of at least three of these criteria [3]. 

Although the exact mechanisms involved in the development of frailty are not yet well understood, in recent years, different etiologic factors have been identified to contribute to this phenomenon. The epigenetic and genetic background, as well as environmental conditions seem to have a relevant role [5,26,27,28]. 

Frailty is often associated with aging or age-related diseases, sharing many mechanisms such as senescence, stem cell exhaustion, hormone dysregulation, DNA damage, mitochondrial dysfunction, oxidative stress, and the loss of proteostasis. Inflammatory processes are proposed to play a pivotal role in the pathogenesis of frailty, suggesting that individuals with rheumatic diseases may have an increased risk of developing this condition [27,29,30]. Chronic low-grade inflammation, known as “inflammaging”, caused by inadequate immune system regulation during the aging process, is considered one of the primary pathogenetic mechanisms of frailty [29,31]. Inflammation’s adverse effects on multiple organ systems, such as loss of strength, reduced physical activity, anemia, clinical and cardiovascular illnesses, and poor nutrition, can lead to frailty directly or indirectly [5,32,33]. 

The association between frailty and high total white cell count [34], particularly neutrophil, monocyte, and CD8+CD28-lymphocyte counts, has been observed [35,36], and an imbalance in the production of pro- and anti-inflammatory mediators is thought to be a driving factor. Indeed, results derived from the Women’s Health and Aging Studies (WHASs) I and II, two population-based studies designed to evaluate the causes and course of physical disability in community-dwelling older women, demonstrated a significant positive association between total white blood cell (WBC) count and frailty, indicating significant risk gradients for frailty even within the normal range of total WBC counts [34]. Furthermore, both neutrophil and monocyte counts had positive cross-sectional associations with being pre-frail and frail. The identified associations were independent of interleukin (IL)-6, albeit the serum levels of this cytokine were higher in these patients [35]. Interestingly, a case–control study of T cell subsets performed in subjects selected from the same cohort revealed that CD8+CD28− lymphocyte counts were significantly higher in frail women when compared with pre-frail and non-frail women [36].

C-reactive protein (CRP), tumor necrosis factor (TNF)-α, and IL-6 are the most-studied molecules linked to muscle catabolism in this context [37,38]. The Longitudinal Aging Study Amsterdam (LASA), a prospective cohort study on predictors and consequences of changes in physical, cognitive, emotional, and social functioning in older people in the Netherlands, demonstrated that moderately elevated levels of CRP were associated with incident frailty [37]. A meta-analysis of cross-sectional studies comparing the inflammatory profile of frail and pre-frail individuals with robust subjects indicated that frailty and pre-frailty are associated with increased CRP and IL-6 serum levels [39,40]. Both frail and pre-frail participants also showed significantly higher serum levels of TNF-α, WBC, and fibrinogen [39]. Other studies have reported the overexpression of pro-inflammatory cytokines, particularly TNF-α and IL-6, in individuals with an increased risk of morbidity and mortality, proposing these levels as markers of frailty [41,42]. Increased IL-6 levels were observed also in an in vitro research study that compared lipopolysaccharide (LPS)-induced cytokine release by peripheral blood mononuclear cells isolated from frail older adults [43]. In addition, reduced production of anti-inflammatory cytokines, such as IL-10, appears to predispose to frailty development. Indeed, it has been observed that subjects genetically predisposed to produce low levels of IL-10 had a higher risk of developing frailty, and IL-10-deficient mice developed biological and clinical features of frailty, including muscle weakness and high IL-6 levels [44,45]. Interestingly, a significant association was also observed between frailty and high plasma levels of high-temperature requirement serine protease A1 (HtrA1) [46]. This is a serine protease involved in the inhibition of the signaling of transforming growth factor-β (TGF-β) 1, an anti-inflammatory cytokine that seems to play a role in healthy aging and longevity [47].

Low-grade inflammation is of pathogenic importance in the development of degenerative rheumatic diseases, such as osteoarthritis (OA) [48,49]. This is a common chronic and disabling condition that affects mainly middle-aged people worldwide, whose exact pathophysiological mechanisms still remain unknown. It is increasingly recognized that joint trauma, mechanical stress, and age-related processes play a part in the disease development and trigger immune-neuroendocrine dysregulation and immunological events, which can lead to joint destruction [50]. In particular, enhanced leukocyte infiltration in the synovium, the presence of active macrophages in synovial fluid, and the production of soluble inflammatory factors including cytokines, chemokines, adipokines, neuropeptides, and metalloproteinases have been recognized to induce and/or contribute to cartilage damage [51]. Increased serum levels of IL-6 and CRP and alterations in HtrA1 expression have been reported in both conditions [52,53]. Multiple mechanisms can lead to frailty in patients with OA. First, patients with lower extremity OA, particularly of the hip and of the knee [54], are more likely to restrict their physical activity, resulting in muscle mass loss and an increased risk of falling than healthy subjects. OA-related pain can contribute to reduced physical activity, thus leading to increased vulnerability. Furthermore, as the incidence of OA increases with age, the frail phenotype can often coexist with the disease, becoming a negative prognostic factor [55]. This could be further influenced by the presence of sarcopenia, a progressive and generalized loss of skeletal muscle mass and strength [56], whose prevalence is increasingly recognized as a correlate of aging and has been observed to be high in knee OA in patients [57].

The link between frailty, rheumatic disorders, and sarcopenia is even more evident in immune-mediated rheumatic diseases, such as rheumatoid arthritis (RA), psoriatic arthritis (PsA), ankylosing spondylitis (AS), systemic lupus erythematosus (SLE), systemic sclerosis (SSc), and vasculitis [57,58,59,60,61], where chronic inflammation facilitates muscle catabolism and weakening of the immune system [62]. Frailty features, such as sarcopenia, fatigue, and low physical activity are prevalent in RA patients [5]. Disease activity in RA is the primary factor in the development of frailty [5,58].

The typical symptoms of frailty, such as weakness, slowness, reduced activity, and low energy, are also present in patients with fibromyalgia (FM), a chronic syndrome that can cause widespread body pain. In this condition, frailty has been proposed as a prognostic marker in the early phases of the disease and could be used to identify potential disability and augmented risk of adverse outcomes [63]. 

Frailty in SLE has been linked to increased mortality, and this association persists even after accounting for potential confounding factors such as age and physician-reported SLE-associated damage. Moreover, frailty has been associated with self-reported disability, independent of age and self-reported SLE disease activity and damage. These findings indicate that frailty is an independent risk factor for significant health outcomes in SLE [64].

Furthermore, different studies reported that higher risks of frailty are related to hormonal changes, such as a decrease of sex steroid levels and an increase of cortisol levels, as well as vitamin D metabolism impairment, which occur with aging and play a direct role in osteoporosis etiopathogenesis [2,65,66].

Finally, frailty in rheumatic diseases can be related not only to the disease itself, but also to the treatment with glucocorticoids and/or with disease-modifying antirheumatic drugs (DMARDs), such as in RA and SLE [5]. DMARDs are essential for steroid-sparing, but immunosuppression can increase infection risk, leading to hospitalization and further risk of frailty [5].

## 4. Spa Therapy Effects on Frailty in Rheumatic Diseases: Immunological Aspects

Currently, there is no standard of care for frailty, and evidence supporting interventions and strategies to reverse or minimize frailty varies across the studies, with a high level of evidence lacking. Interventions for frailty aim to prevent, postpone, reverse, or lessen its consequences [67,68]. Spa therapy, the use of natural mineral waters and peloids, is a widely used non-pharmacological intervention to treat rheumatic diseases, which has been shown to be beneficial in symptom reduction and is considered by the Osteoarthritis Research Society International (OARSI) guidelines an appropriate treatment for patients with multi-joint OA and comorbidities [68,69,70,71,72]. To date, very few studies have been conducted on the direct effect of spa therapy on frailty, and the cellular and molecular mechanisms that might be involved have never been investigated. Consequently, the effect can only be deduced from studies on rheumatic diseases or on the risk factors that lead to frailty. Different mechanisms of actions combining thermal, mechanical, chemical, and microbiological factors have been identified for spa therapy in several rheumatic diseases [15,16]. In particular, its immunomodulatory properties seem to play a key role in treatment efficacy [14,73], thus suggesting its potential effectiveness in the regulation of inflammaging in frailty. 

The effects of spa therapy have been most studied in OA, where anti-inflammatory, antioxidant, and chondroprotective activities have been demonstrated. Indeed, several in vitro studies on cultured chondrocytes or synoviocytes from OA patients have shown that hydrogen sulfide (H_2_S) donor treatment is able to modulate mitogen-activated protein kinase/extracellular-signal-regulated kinase (MAPK/ERK) and nuclear factor kappa-light-chain-enhancer of activated B cells (NF-kB) pathway activation, reducing proinflammatory mediator secretion involved in OA, such as IL-6, IL-8, nitric oxide (NO), prostaglandin E2 (PGE2), and matrix metalloproteinases (MMPs) [73,74]. In clinical trial, besides a reduced serum concentrations of pro-inflammatory molecules, including IL-6, TNF-α, IL-1β, IL-8, PGE2, leukotriene B4 (LTB4), and extracellular heat shock protein (eHsp72), and the expression of microRNA associated with cartilage degradation, augmented levels of circulating cortisol have been found in OA patients after spa therapy [74,75,76,77,78]. Recently, a study on hemorheological blood indices of patients with OA revealed that sulfur balneotherapy improved erythrocyte deformability and aggregation parameters, along with a reduction of the neutrophil number, without any significant changes in fibrinogen and CRP levels [79]. Interestingly, poor deformability has been observed in senescent erythrocytes, which may contribute to frailty onset and progression [80]. Although there is no consensus on the use of spa therapy in the treatment of patients with inflammatory rheumatic diseases, a reduction of IL-6, TNF-α production, and other inflammatory processes has been reported in in vitro models of RA in the presence of H_2_S donors [14]. Moreover, spa treatments through mud bath applications have been observed to decrease inflammation in rats with arthritis induced by the subplantar injection of Freund’s complete adjuvant or by zymosan intra-articular injection [81,82]. Of interest, the anti-inflammatory effects of spa therapy have been demonstrated in patients with chronic back pain, which reported a significant increase in circulating anti-inflammatory cytokine IL-10, a decrease in IL-6 serum levels, and changes in the serum content of proteins involved in several functions, including tissue repair, angiogenesis, and modulation of gene expression [83,84]. Despite only a few studies on the effects of spa therapy having been carried out in seronegative spondyloarthritis, clinical trials have shown a reduction in the serum concentration of pro-inflammatory cytokines and an enhancement in the total and active circulating TGF-β1 in patients with AS after treatment [85,86]. 

Spa therapy has been confirmed as beneficial for FM syndrome (FMS), where it has demonstrated anti-inflammatory and antiaging activity. FMS patients receiving mineral-rich water bathing reported a decrease in PGE2, IL-1, and LTB4 serum levels [87], while mud treatments revealed a negative correlation between the telomere length of peripheral leukocytes and red blood cell counts or serum albumin, thus suggesting the possibility of extending the life-span of circulating leukocytes [88]. However, no significant changes have been detected in cortisol and dehydroepiandrosterone sulfate (DHEA-S) serum levels in FMS patients after balneotherapy [89]. Anti-inflammatory effects, accompanied by proliferative and antioxidant properties, have been reported also in cultured human osteoclasts and murine osteoblasts after treatment with H_2_S donors [14], thus suggesting that spa therapy may improve the bone damage induced by osteoporosis. This has been confirmed in a rat model of subchronic inflammation, where a cold mud bath demonstrated a beneficial effect on bone turnover metabolism and resistance to fracture [90]. 

Of note, the effects that spa therapy could exert on risk factors for rheumatic diseases and frailty, such as overweight, should not be overlooked. For instance, it has been demonstrated that spa therapy induced a reduction in body weight, total cholesterol, low-density lipoprotein (LDL) cholesterol, triglycerides, glycemia, and CRP and leptin serum levels, but an increased in adiponectin, in obese subjects [91,92]. Furthermore, a comprehensive health education program combining lifestyle education and hot spa bathing has revealed that participants in the study maintained their immunological function and reported a decrease in body fat percentage [93].

## 5. Spa Therapy Effects on Frailty in Rheumatic Diseases: Clinical Evidence

### 5.1. Spa Therapy for OA Patients

Traditional spa therapy interventions have several positive effects in rheumatic patients. Numerous studies have shown that, in patients with OA, a cycle of baths in thermal mineral-rich water can improve motor function and pain, considerably more than immersion in tap water [81,93,94,95,96,97,98]. 

In a randomized, double-blind study on 58 patients with knee OA, balneotherapy delivered as a 15-day course of 30 min daily sessions performed with sulfur water from Cserkeszölö in Hungary demonstrated a significantly greater improvement in pain, range of motion, tenderness on palpation, functionality, and subjective rating when compared with immersion in tap water [99]. Twelve daily sulfate-bicarbonate-calcium mineral water baths demonstrated long-lasting beneficial effects in patients with knee OA, including alleviation of pain symptoms, improved functional capacities, and enhanced quality of life, even at a follow-up evaluation after 12 weeks [98].

In a randomized controlled trial, the treatment group underwent 30 sulfurous baths over a 10-week period at a temperature of 37-39 °C. The study results showed significant improvements within the treatment groups in the pain scores, reduced use of pain medication, and enhanced functional status, even two months after the intervention, indicating the long-lasting effects of sulfurous baths [97]. 

Furthermore, treatments in Dead Sea sulfur water have been shown to be effective in ameliorating patients’ conditions. In 42 patients with knee OA who were treated twice weekly for six consecutive weeks in a sulfur pool at 35–36 °C, OA severity, quality of life, and pain demonstrated a statistically significant improvement, lasting up to 6 months, when compared to a control group treated in tap water [100]. Similar effects on pain and motor function were also described in patients with hand and spine OA treated with sulfur water [101,102]. 

A recent systematic review evaluated balneotherapy’s effects in OA. The findings of all the studies included in the analysis indicated an improvement in all the symptoms and signs examined. Notably, pain and quality of life were the primary symptoms assessed, and both demonstrated improvement following treatment with thermal mineral-rich water in all the included studies. Nevertheless, it is important to note that the quality of several studies was deemed suboptimal, highlighting the need for further clinical trials in this field [103].

In knee OA, also mud therapy has been shown to be an appropriate choice for pain relief and functional improvement [103,104,105,106,107,108,109,110,111]. In a study, a total of 80 patients with knee OA were divided into three groups: balneotherapy, mud-pack therapy, and hot-pack therapy. The therapies were administered once a day for 20 min, five times a week, for a total of 10 sessions. The results showed statistically significant improvements in pain scores for all three groups, while quality of life showed significant improvements in the balneotherapy and mud-pack therapy groups, while the hot-pack therapy group did not show significant changes [112]. The combination of balneotherapy and peloid therapy has also been shown to be effective [112,113,114,115], with a reduction of symptomatic drug consumption and the persistence of the benefits 9 months after the end of the treatments [113,116,117]. 

### 5.2. Preventive and Rehabilitative Strategies in the Spa Setting for OA Patients

The addition of rehabilitative treatments to traditional spa interventions has been demonstrated to further contribute to the benefits regarding pain and motor function in OA patients [118,119,120,121,122]. A spa-rehabilitation program (12 sessions of stretching and strengthening exercises, floating or standing in the thermal pool) was found to have a clear clinical benefit on patients with chronic back pain secondary to axial OA in terms of pain and disability reduction [84]. In another study involving the enrollment of 160 patients with degenerative musculoskeletal disorders from six Italian spa facilities, all the participants underwent 12 sessions of water-based exercise, including joint exercises, muscle strengthening, gait training, and proprioceptive and balance techniques, conducted in thermal mineral-rich water or warm water pools. Additionally, some patients received traditional spa therapies such as mud therapy and thermal baths. Overall, patients experienced positive effects on pain, mood, and quality of life through water exercise training alone or in combination with traditional spa therapy [123]. A recent study conducted in the Austrian Alps demonstrated significant pain improvements in patients with various rheumatic conditions, including knee and hip OA. The patients, after undergoing low-dose radon treatment, physical exercise, massages, lymphatic drainage, mud therapy, back pain and anti-smoking prevention consultations, and healthy nutrition maintained the positive effects of the intervention for up to 9 months [124].

### 5.3. Spa Therapy for RA Patients

In RA, spa therapy added to the usual pharmacotherapy was demonstrated to reduce disease activity [125,126], which appears to be a factor linked to an increased risk of developing frailty [5]. Bathing, either alone or when combined with Dead Sea mud packs, has been proven effective in reducing pain intensity and arthritis impact [127,128]. Positive effects on the quality of life of RA patients have been recorded for both mineral bathing and immersion in sand or mud [111,129]. Radon and carbon dioxide baths in addition to daily specific exercises have been demonstrated to be beneficial in reducing corticosteroid, NSAID, and/or analgesic consumption [96], another factor hypothesized to underlie the risk of developing the frail phenotype.

### 5.4. Preventive and Rehabilitative Strategies in the Spa Setting for RA Patients

Recently, a multimodal, non-pharmacological spa therapy intervention including low-dose radon treatment showed significant improvements in pain in rest and motion until 9 months after the therapy. This study considered patients with different rheumatic conditions, including RA. The comprehensive intervention encompassed a range of treatments and therapies aimed at promoting physical and mental well-being. It included various components such as low-dose radon balneotherapy and/or low-dose radon speleotherapy, physical exercise, mud therapy, healthy nutrition, and prevention consultations [124]. 

### 5.5. Spa Therapy for FM Patients

Positive effects on pain, disease impact, and fatigue have also been demonstrated in FM patients with a cycle of thermal baths and peloid applications [130,131,132,133] with greater short- and long-term improvements than usual medical treatment and/or daily exercises [134]. An immediate versus a delayed spa therapy was evaluated in a 12-month randomized clinical trial, demonstrating a clinically significant improvement at 6 months that persisted for 12 months in those who performed the immediate spa therapy [135]. Furthermore, balneotherapy and spa therapy can lead to increased quality and quantity of sleep, by affecting some hormones such as histamine and serotonin and regulating body temperature [135,136,137,138].

### 5.6. Preventive and Rehabilitative Strategies in the Spa Setting for FM Patients

In a randomized controlled trial, an FM-specific therapeutic education program added to spa therapy showed benefits in quality of life and pain intensity compared to standard therapy alone [139]. At 6 months, the physical exercises taught during the education program were still practiced regularly by 87% of patients [139]. Furthermore, the combination of low-dose radon treatment, physical exercise, massages, mud therapy, prevention strategies, education strategies and healthy nutrition demonstrated some beneficial effects in FM patients [124].

### 5.7. Spa Therapy for SLE Patients

A study aimed to assess the effects of balneotherapy on non-inflammatory complaints, quality of life, and work productivity was conducted in patients with SLE who were in remission or had low disease activity. The study included thirty SLE patients and divided them into two groups: one receiving balneotherapy in addition to standard care and the other receiving standard care only. The results showed that the group receiving balneotherapy experienced significant improvements in physical condition, general health, and several subdomains of physical health based on the 36-Item Short Form Survey (SF-36) questionnaires [140]. 

### 5.8. Other Effects 

Besides playing an important role in the treatment of rheumatic diseases, the spa setting appears to be appropriate for managing subjects suffering from comorbidities, which often overlap with frailty [3,4,141,142,143]. For instance, exercise in thermal mineral-rich water seems to provide the safest environment for rehabilitating obese rheumatic patients, who can benefit from load reduction due to the water buoyancy force, minimizing the risk of joint injury and enabling more extensive movements [70]. Additionally, the combination of aquatic exercise and education can be useful in achieving and sustaining significant weight loss results [144,145]. Thermal water exercise also appears to have favorable effects on psychological comorbidities. Bathing in water with varying mineral content has been shown to contribute to reducing depression in FM patients [146,147,148] and could positively influence anxiety and mood states [97]. The combination of balneotherapy, other spa treatments, and physical exercise also appears to be effective in enhancing self-perceived sleep quality [138].

In Table 1, all the effects found in clinical and preclinical studies are summarized.

## 6. Discussion

Frailty is defined by a deterioration in various systems’ physiologic reserve and function, which increases susceptibility and has detrimental effects on health [3]. Recent research has highlighted the higher frequency of frailty in chronic diseases, regardless of age, even though it is widespread in the elderly [5]. Chronic inflammation is likely to be crucial in frailty development, both directly and through the effects on other systems such as the musculoskeletal, endocrine, and neurological systems [58]. Frailty is more common in rheumatic disease patients than in healthy controls, regardless of age, and is linked to increased disease activity, mostly because of chronic inflammation [5]. For example, since the incidence of OA increases with age, the frail phenotype can often coexist with this disease, also associated with sarcopenia [5]. The link between frailty, rheumatic disorders, and sarcopenia is even more evident in immune-mediated rheumatic diseases, such as RA, AS, and SLE, where chronic inflammation facilitates muscle catabolism [5]. The typical symptoms of frailty, such as weakness, reduced activity, and low energy, are also present in patients with FM [63]. 

Frailty is not a permanent condition, and early interventions should be attempted to prevent it while also limiting disabilities and severe health outcomes [2,67]. Exercise training has demonstrated positive effects on various systems, including the musculoskeletal, endocrine, and immunological systems. In particular, exercise helps to reduce frailty by suppressing muscle inflammation, promoting anabolism, and increasing muscle protein synthesis [67,149,150]. Combined aerobic and resistance exercise seem to be the most-effective strategies to ameliorate frailty condition [150]. As mentioned above, muscle strength is one of the predictive characteristics associated with frailty, as well as one of the elements impacted in rheumatic diseases such as osteoporosis, OA, FM, and RA. Progressive resistance training has been shown to increase muscle mass and gait speed in older adults and can, therefore, be considered an effective rehabilitative intervention for frail subjects [150]. On the other hand, a reduction in aerobic capacity has been shown to be linked to frailty and aging, contributing to a decrease in the ability to perform the activities of daily living [50,150]. 

Water-based rehabilitation is a widely employed approach for the prevention and management of numerous conditions [14,103,111,122,151]. In the field of musculoskeletal conditions, water-based exercise has been utilized to enhance pain management and functional outcomes. On the other side, in the case of neurological disorders, there is notable evidence supporting aquatic exercise effectiveness, particularly in Parkinson’s disease and strokes [122,152]. These conditions can benefit from water-based rehabilitation, as it helps reduce the risk of falls and enhances exercise safety [122]. Additionally, water-based rehabilitation plays a significant role in cardiopulmonary rehabilitation. Indeed, water-based exercise has been shown to improve respiratory and peripheral muscle strength [122].

Exercise in thermal mineral-rich water combines the benefits of hot mineral waters, such as the pharmacological effects and the physical effects of immersion (mainly due to temperature, buoyancy, viscosity, and hydrostatic pressure), with the already-mentioned effects of exercise [14,16,143,153]. Exercise programs conducted in the spa setting can include exercises to maintain and improve joint function, mobility, and flexibility and strengthening exercises [121]. Indeed, the warm temperature of the water promotes relaxation and reduces muscle tension, thereby alleviating pain and enhancing mobility. The buoyancy provided by the water reduces the load on joints, facilitating gentle exercise and promoting functional recovery. These improvements are of great significance in the context of frailty, as they contribute to enhanced mobility, reduced pain, and improved overall well-being. Furthermore, proprioception exercises and gait training can also be performed in the spa setting [10,152,154,155,156]. 

On the other side, also traditional spa therapy interventions alone have several positive effects in rheumatic patients. The reviewed studies consistently reported improvements in pain, physical function, and quality of life following spa therapy interventions. The mineral content contributes to spa therapy’s positive effects by offering anti-inflammatory and analgesic properties. H_2_S donor treatments have shown promise in reducing inflammation and proinflammatory mediators associated with OA. Although the use of spa therapy in inflammatory rheumatic diseases is still debated, in vitro models of RA have shown reduced production of IL-6 and TNF-α. In the case of FM patients as well, mineral-rich water bathing has displayed anti-inflammatory effects, leading to decreased serum levels of PGE2, IL-1, and LTB4. 

In addition to its effects on specific conditions, spa therapy has shown promise in addressing risk factors for rheumatic diseases and frailty. For instance, spa therapy has been associated with reductions in body weight, cholesterol, triglycerides and glycemia levels in obese individuals. Moreover, spa therapy interventions often incorporate multidisciplinary approaches, including physical exercises, social integration strategies, healthy nutrition, and neuropsychologic sessions [11,124,157,158]. These holistic approaches address various aspects of frailty, including physical, psychological, and social domains. 

Beyond the specific treatments, the tranquil environment of a spa facility contributes significantly to the therapeutic effects [10,14,123]. Spa therapy often takes place in serene settings, away from the stresses and distractions of daily life. This tranquil setting helps to reduce anxiety, improve sleep quality, and enhance mental well-being [76,123,138,159]. 

Due to all these beneficial effects, the exploration of preventive and rehabilitative protocols in the spa setting is currently an emerging field. Previous studies have evaluated a multidimensional approach in the spa setting to improve patients’ quality of life and promote their well-being through a combination of physical, nutritional, and educational elements [11,124]. Similar to the results obtained in the treatment of rheumatic conditions, preliminary findings indicate that a tailored and multidisciplinary rehabilitative program, incorporating thermal mineral-rich water exercises, respiratory and motor exercises, social integration training, neuropsychology sessions, LASER therapy, and magnetotherapy, has promising results in reducing the exacerbation of long COVID syndrome, preventing the onset of disabilities, and minimizing reliance on medications and specialist consultations [157,158].

Based on the results of our scoping review, the spa setting holds significant potential for implementing preventive and rehabilitative protocols tailored to frailty patients with rheumatic disorders. Preventive protocols in the spa setting can focus on early intervention and lifestyle modifications to mitigate the progression of rheumatic disorders and reduce the risk of frailty development. These protocols may include personalized exercise programs that incorporate aquatic therapy sessions aimed at improving joint flexibility, muscle strength, and overall physical function. In addition, nutritional counseling and education strategies can be incorporated to promote healthy habits and optimize well-being. 

The main limitation of our review is the lack of direct investigation into the effect of spa therapy on frailty itself. As stated, there have been very few studies specifically examining the impact of spa therapy on frailty, and no research has explored the underlying cellular and molecular mechanisms involved. Therefore, the understanding of the effect of spa therapy on frailty is primarily based on extrapolating findings from studies conducted on rheumatic diseases or the risk factors associated with frailty. This indirect approach limits the ability to draw definitive conclusions about the direct effects of spa therapy on frailty and highlights the need for further research specifically targeting this area.

## 7. Conclusions

In conclusion, by contributing to the body of knowledge on non-pharmacological treatments for frailty, this review provided a comprehensive understanding of the effectiveness of spa therapies in managing frailty in rheumatic patients. The results of various in vitro, in vivo, and clinical studies on pain, disease activity, function improvement, comorbidity management, and inflammation modulation suggest that spa treatments may be effective in preventing frailty in rheumatic patients. Furthermore, the health resort setting’s unique benefits make it an ideal environment to develop specific exercise programs for patients with frailty, rheumatic diseases, and comorbidities. Therefore, future studies should investigate innovative strategies to improve the tailored and comprehensive management of these patients in the spa setting.

## Figures and Tables

**Table 1:** Effects of spa therapy on rheumatic conditions.

**Anti-inflammatory and immunomodulatory effects**	Modulation of MAPK/ERK pathway and NF-kB pathway activation
	Reduction of IL-6, IL-8, NO, PGE2, and MMPs
	Reduction of TNF-α, IL-1β, LTB4, and eHsp72
	Increase in circulating anti-inflammatory cytokine IL-10 and TGF-β1
**Cartilaginous effects**	Reduction of the expression of microRNA associated with cartilage degradation
**Skeletal effects**	Beneficial effect on bone turnover metabolism
**Endocrinological effects**	Reduction in body weight, body fat percentage, total cholesterol, LDL cholesterol, triglycerides, glycemia, and CRP and leptin serum levels
	Increase in adiponectin, in obese subjects
**Motor effects**	Improvement of motor function
	Reduction of stiffness and muscle tension
**Pain effects**	Reduction of perceived pain
	Reduction of symptomatic drug consumption
**Disease activity effects**	Reduction of disease activity in RA patients
**Psychological effects**	Increase in levels of circulating cortisol
	Favorable effects on psychological comorbidities
	Increase in sleep quality

MAPK/ERK: mitogen-activated protein kinase/extracellular-signal-regulated kinase; NF-kB: nuclear factor kappa-light-chain-enhancer of activated B cells; IL: interleukin; NO: nitric oxide; PGE2: prostaglandin E2; MMPs: matrix metalloproteinases; TNF-α: tumor necrosis factor; LTB4: leukotriene B4; eHsp72: extracellular heat shock protein 72; TGF-β1: transforming growth factor-β1; LDL: low-density lipoprotein; CRP: C-reactive protein.

## Data Availability

The literature review was conducted using the MEDLINE (PubMed), PEDro, and Scopus databases.
